# Supplementary material for: Promoting the health and wellbeing of children: A feasibility study of a digital tool among professionals
Source: PLoS One. 2022 Mar 22;17(3):e0265355. doi: 10.1371/journal.pone.0265355 (PMC8939777; doi:10.1371/journal.pone.0265355)
Supplement: Attachment — Submitted filename: Manuscript.docx [file pone.0265355.s003.docx]

Promoting the health and wellbeing of children: a feasibility study of a digital tool among professionals

Magda Skogberg^1¶^, Karolina Mackiewicz^2&^, Kristel Mänd^3&^, Lehte

Tuuling^3&^, Indra Urdzina-Merca^4&^, Sanna Salanterä^1, 5&^ and Anni

Pakarinen^1¶^*

1 Department of Nursing Science, University of Turku, Turku, Finland
2 European Connected Health Alliance, Helsinki, Finland
3 Rakvere City Government, Rakvere, Estonia
4 Welfare Department, Jurmala City Council, Jurmal, Latvia
5 Turku University Hospital, Turku, Finland

*Corresponding author

Email: anni.pakarinen@utu.fi (AP)

^¶^ These authors contributed equally to this work.

^&^These authors also contributed equally to this work.

# Abstract

The foundations of children’s health and wellbeing are laid in early childhood. A gamified app (EmpowerKids tool) was designed to support professionals to have discussions with 6- to 12-year-olds from low-income families about their health and wellbeing. The aim of this feasibility study was to evaluate the usability and acceptability of the tool from the perspective of professionals in social, health and education settings. The study was conducted using a one-group post-test-only design. The usability data were collected using System Usability Scale and the acceptability data were collected using an open-ended questionnaire distributed to professionals (n = 24) in Estonia, Finland and Latvia. The data were collected during two phases. The tool was modified further on the basis of the results. The total usability scores were 82/100 (first testing) and 84/100 (second testing), indicating excellent usability. The answers related to acceptability were divided into four categories: suitability for the context; satisfaction and quality; attractiveness; modification needs. The professionals perceived that the tool helped them to build an overall picture of a child’s health and wellbeing, and to gain information about the child’s individual needs. The requirements for modification detected during the first testing were mostly related to difficulties with textual expressions and graphics. No major modification requirements were expressed during the second testing. The tool is considered feasible and may be used by professionals from different settings to support children’s health and wellbeing. Further studies are needed to evaluate the effectiveness of the tool from the perspective of child outcomes.

**Keywords:** children; wellbeing; health promotion; gamification; feasibility

# Introduction

Childhood is a sensitive formative period of a person’s life, during which the socioeconomic status of their parents can have immediate and long-lasting adverse effects on the person’s health and wellbeing [1]. Health inequalities and health problems in adulthood are more common among people who have grown up in a low-income or vulnerable family than for people who are from wealthier families [2, 3]. Low socioeconomic status has also been associated with disparities in health literacy [4, 5]. Health literacy refers to an individual’s capacity to access, process, and appropriately interpret and act on essential health information and services to make health-related decisions [6, 7]. Subsequently, low health literacy is related to poorer health behaviors [8] and worse health outcomes [9]. Related term, mental health literacy refers to individual’s knowledge and beliefs about mental health disorders that help in their recognition, prevention and management [10, 11]. Low levels of both health literacy types correlate with poorer mental [11, 12] and physical health, more use of healthcare than preventive care, and increased mortality [13].

Although, parents’ decisions on health-related issues in the family will affect a child’s health and wellbeing, children are also entitled to influence matters that concern their own life [14]. A child’s wellbeing can be seen as a multi-dimensional construct, which consists of mental, physical and social dimensions [15]. Health behaviors, such as those relating to physical activity [16] and nutrition [17], in addition to family and personal resources [18, 19] and a balanced daily rhythm [20, 21], are factors that have an effect on a child’s health and wellbeing. The foundations of a person’s health, wellbeing and life success are laid in early childhood [22]; therefore, efforts to promote health and wellbeing should be targeted at this period of life [23].

Professionals working with young children in health and social care or educational settings are in a key position to promote health and wellbeing. During interventions, it is fundamental to empower a child’s active participation [24] and increase the child’s knowledge, motivation, competence and self-efficacy. Supporting an individual’s ability to make health-promoting decisions in their own life may have a significant influence on their overall health and wellbeing in the long term [25]. Methods of promoting the health and wellbeing of children should be efficient and evidence-based [26]. The purpose should be to achieve optimal health and wellbeing, and to remove health inequalities; for example, by ensuring equal opportunities and resources for everyone [27]. These methods should also be child-centered, age-appropriate [28] and implemented in a way that supports the child’s health literacy [8, 29].

Schools offer an optimal setting for interventions among children and digital interventions seem to attract children from digital age [30]. According to a recent review in school-based health promotive interventions, effective interventions included digital components, such as internet-based counselling, text messages, websites and social media. Results showed that these digital interventions often included also other components, such as face-to-face discussions with teachers and interventions addressed to parents [31]. Many of previous digital interventions address children’s health behaviors, but there seems to be lack of digital interventions addressing child’s mental, social health and wellbeing [30].

Health games can provide child-friendly and effective ways of promoting health [32,

33] and health education [34, 35]. Evidence suggests that health games and gamified apps have positive effects on children’s health and wellbeing [36], their health behaviors [37, 38] and their health-related knowledge [39]. Despite being evidence-based, however, to be effective health games and gamified apps also need to be motivating, engaging and entertaining [40]. In addition, the content of the games should be age appropriate and ethically sustainable [41].

This study was conducted as part of a project entitled “EmpowerKids – Health education and social advice for low-income families with small children,” which ran from the year 2016 to 2018 and was co-financed by the Central Baltic Programme 2014 – 2020. The EmpowerKids project focused on tackling social exclusion, inadequate health information and inadequate advice provided by social care services among children in low-income families in Estonia, Finland and Latvia. The project aimed to develop an intervention that would support professionals in promoting the health and wellbeing of children [42].

Given that games and apps are an integral part of children’s everyday life, a gamified, Internet-enabled app (the EmpowerKids tool; also referred to as “the tool”) was designed to support professionals in discussions with individual children about their health behaviors, physical activity and nutrition, family and personal resources, and daily habits. The EmpowerKids tool was developed at the University of Turku in collaboration with health professionals, social workers and preschool teachers from Estonia, Latvia and Finland [42, 43]. The development followed an iterative user-centered design process [44], which included five phases (Fig 1). Phases three and four cover the feasibility study reported in this article.

**Fig 1. Iterative user-centered design process.**

Feasibility studies are important in the development and testing of an intervention. A feasibility study explores the relevance, sustainability and modification requirements of an intervention. In general, such studies have different areas of focus (e.g., acceptability) depending on the intervention and the aim of the study. The chosen area of focus determines the optimal research design. [45, 46]

The aim of this feasibility study was to evaluate the usability and acceptability of the EmpowerKids tool from the perspective of professionals in social, health and education settings.

# Materials and methods

## Study design

The study used a one-group post-test-only design [47]. The data were collected during the feasibility testing in February 2017 and again in September 2017 in order to examine professionals’ opinions about usability and acceptability of the EmpowerKids tool (Fig 2).

**Fig 2. Study design.**

### Intervention

The target group for the intervention consisted of 6- to 12-year-olds from low-income families, and the professionals who were working with these children in social, health and educational settings. The intervention had two phases. First, a child entered information into the tool about their own health behaviors, their family and personal resources and their daily habits. Second, a health discussion took place between the child and a professional. The discussion with the child was based on the results provided by the tool for the child’s physical activity, nutrition, family and personal resources and daily habits. The professionals were given a 15-hour training session about the intervention before they used it.

The tool was designed with two user interfaces: one for the child (Fig 3) and one for the professional. It consisted of four sections: physical activity, nutrition, family and personal resources and daily rhythm. In the physical activity and nutrition sections (Fig 4), the child made choices based on their personal health behaviors by picking suitable apples (containing alternatives) for the baskets. In the daily rhythm section, the child made choices based on their personal habits (e.g., hygiene routines, hobbies, meeting friends), by picking suitable apples (containing alternatives) for the baskets. The baskets stood for the frequency of the habit (“every day,” “a few times a week,” “once a week,” “sometimes”). In the resources section, the child scored different statements (n = 15) by choosing facial expressions that were based on a 4-point Likert scale (from “I agree” to “I disagree”). The children were instructed to choose a face (from a smiley face to a sad face) that best described their level of agreement with each statement (e.g., “I feel safe,”

“I feel good at home,” “I enjoy learning”) by expressing strong or mild agreement or disagreement. The scale was developed based on previous literature covering subjective and objective aspects of child wellbeing [e.g., 48, 49, 50] and involved experts in health research along with professionals working with children from the fields of health, social work and education. The set of statements included was proposed to represent the physical, emotional/psychological, social, educational and economic wellbeing of the child (Fig 5).

**Fig 3. Children’s interface of the EmpowerKids tool.**

**Fig 4. Nutrition section of the EmpowerKids tool.**

**Fig 5. Resources section of the EmpowerKids tool**

After completing each section, the children received visual feedback on their results. Feedback related to health behaviors was based on international [51] and national recommendations (i.e., current nutritional recommendations for children in each country).

The professionals also received the feedback on each child’s results (Fig 6).

**Fig 6. Feedback for healthy nutrition.**

They could then use the visual feedback in their discussion with the child. The tool’s database allowed for the collection of metadata about children’s health and wellbeing, which can be used, for example, to guide the planning of activities and interventions in an organization.

## Study sample and recruitment

The study participants were recruited during the EmpowerKids project and the data were collected using a purposive sampling method. There was no predetermined sample size, since feasibility studies are not expected to have large sample sizes to show statistically significant findings. Rather, the aim was to gain understanding about the intervention in real-life settings with various people that best represent the intended group of end-users. [52, 53] All the eligible professionals, attending to the intervention and using the tool with children, were recruited to the study. Professionals were given the information letter and informed consents were acquired. All together 14 professionals in the first testing phase and 10 in the second testing phase, voluntarily participated in the study. The study participants were professionals working with children with different roles in the organization.

During the first phase of testing (February 2017), the tool was tested in three countries (Estonia, Finland and Latvia) by children ranging from 6 to 12 years old (n = 95) and professionals working with these children in childcare (n = 11) and basic education settings (n = 3), with different roles in the organizations; manager (n = 1), head of the education (n = 1), project coordinator (n = 1), social worker (n = 3), elementary school teacher (n = 3), preschool teacher (n = 3), school health nurse (n = 1) and social pedagogue (n = 1). The study settings included school healthcare (n = 1), kindergartens (n = 4), children’s centers or social centers (n = 5), elementary schools (n = 3) and a city welfare department (n = 1).

During the second phase of testing (September 2017), the tool was tested in three countries (Estonia, Finland and Latvia) by a different set of children ranging from 6 to 12 years old (n = 69) and different professionals working with these children in childcare (n

= 9) and basic education settings (n = 1), with different roles in the organizations; manager (n = 2), head of the education (n = 2), project coordinator (n = 1), social worker (n = 3), elementary school teacher (n = 1), preschool teacher (n = 1). The study settings included kindergarten (n = 2), children center or social center (n = 5), elementary school (n = 3) and city welfare department (n = 1).

## Data collection and outcome measures

The data were collected using the System Usability Scale (SUS) [54] and an open-ended questionnaire after the professionals had used the EmpowerKids tool during their health discussions with children.

### System Usability Scale

The SUS evaluates the subjective assessment of the usability of a specific system [55]. Evidence shows that the SUS [54] is a reliable and valid measure of usability [55]. The SUS is a ten-item Likert scale with five response options that yield the respondents’ level of agreement or disagreement with statements on the usability of the tool. The ten items in the scale alternate, with five positive statements (odd numbers) and five negative statements (even numbers). The score can range from 0 to 100, where a higher number represents a more usable tool [55]. The SUS used in this study was translated into Finnish,

Estonian and Latvian.

### Acceptability of the Tool

A questionnaire was developed especially for this study (Table 1). The questionnaire evaluated the acceptability of the tool and modification needs. The questionnaire consisted of five open-ended questions that assessed the respondents’ opinions about the EmpowerKids tool. The questions covered general opinions on elements of the tool and ideas for further modification of the tool. The respondents were also able to add any other comments they had about using the tool.

**Table 1. Acceptability questionnaire developed for the study.**

| 1 | What did you like about the EmpowerKids app? |
| --- | --- |
| 2 | What did you dislike about the EmpowerKids app? |
| 3 | What do you want to be retained in the EmpowerKids app? |
| 4 | What do you want to be removed or changed in the EmpowerKids app? |
| 5 | Are there any other comments you want to make about the EmpowerKids app? |

### Descriptive statistics

A standard analysis of the SUS scores and the synthesis of the results was performed using a template in Microsoft Excel 2016. The overall SUS score was calculated according to the special calculating system: for each positive answer (statements 1, 3, 5, 7, 9), one point from the user’s response (1 to 5) was subtracted and for each negative answer (statements 2, 4, 6, 8, 10), the user’s response (1 to 5) was subtracted from five. The sum of the scores for all ten items was then multiplied by 2,5, which gave an overall SUS score, ranging from 0 to 100 [55]. Complementary adjective ratings for the SUS scores were also defined to initiate a distinct description of the overall usability of the EmpowerKids tool [56].

### Qualitative data analysis

The responses to the open-ended questions were analyzed after the first phase of testing by performing an inductive content analysis to produce a synthesis of the main findings [57]. The aim was to attain a condensed and comprehensive description of the acceptability of the EmpowerKids tool. During the analysis, we moved from specific to general data, so that specific items were observed and then combined into a general category. The outcome of the inductive content analysis were four generic categories; suitability for the context, satisfaction and quality, attractiveness, and modification needs. The generic categories describe the main categories for which professionals expressed an opinion on the usability and acceptability of the tool. The responses to the same open-ended questions from the second phase of testing were analyzed by performing a deductive content analysis, using the category structure that was created from the previous inductive content analysis. This made it possible to compare the categories between the first and second testing points.

## Ethical considerations

The authors assert that all procedures in the study were performed in accordance with the ethical principles and responsible conduct of research [58, 59]. This study was conducted among adult participants (professionals in the given settings) during a EmpowerKids project. The children and their parents, who were taking part in the project, were informed about the project and consents were acquired from them before start of the project in their schools or other settings. All the eligible professionals were given the information letter and informed consents were acquired. Professionals voluntarily participated in the study.

# Results

## Usability of the EmpowerKids tool

### First phase of testing (version 1)

The overall average usability of the EmpowerKids tool in all countries was good, with an average SUS score of 82 across all countries. The professionals’ average system usability ratings were higher in Latvia (95) than in Estonia (83) and Finland (64) (Tables

2 and 3). For the professionals in Estonia (n = 7), the scores ranged from 62,5 (OK) to

100 (best imaginable). For the professionals in Finland (n = 3), the scores ranged from 60 (OK) to 67,5 (OK). All the professionals in Latvia (n = 4) rated the tool at 95 (excellent).

**Table 2. SUS scores after first testing phase.**

| **Item** | FIN  (n = 3) | | |  |  | EE (n = 7) | | |  |  |  | LV  (n = 4) | | | Mean  (FIN) | Mean  (EE) | Mean  (LV) | Mean  (all) |
| --- | --- | --- | --- | --- | --- | --- | --- | --- | --- | --- | --- | --- | --- | --- | --- | --- | --- | --- |
| 1. I think that I would like to use this app frequently. | 3 | 3 | 3 | 3 | 3 | 3 | 3 | 4 | 5 | 5 | 4 | 4 | 4 | 4 | 3 | 3.7 | 4 | 3.64 |
| 2. I found this app unnecessarily complex. | 4 | 2 | 4 | 1 | 2 | 2 | 4 | 1 | 1 | 1 | 1 | 1 | 1 | 1 | 3.33 | 1.71 | 1 | 1.85 |
| 3. I thought this app was easy to use | 2 | 4 | 4 | 4 | 4 | 4 | 3 | 5 | 4 | 5 | 5 | 5 | 4 | 4 | 3.33 | 4.14 | 4.5 | 4.07 |
| 4. I think that I would need assistance to be able to use this app. | 1 | 2 | 2 | 1 | 1 | 1 | 1 | 1 | 1 | 1 | 1 | 1 | 1 | 1 | 1.66 | 1 | 1 | 1.14 |
| 5. I found the various functions in this app were well integrated. | 4 | 4 | 3 | 3 | 3 | 3 | 4 | 3 | 5 | 5 | 4 | 4 | 5 | 5 | 3.66 | 3.7 | 4.5 | 3.92 |
| 6. I thought there was too much inconsistency in this app. | 3 | 2 | 2 | 1 | 2 | 2 | 3 | 2 | 1 | 1 | 1 | 1 | 1 | 1 | 2.33 | 1.71 | 1 | 1.64 |
| 7. I would imagine that most people would learn to use this app very quickly | 4 | 3 | 4 | 4 | 4 | 5 | 5 | 5 | 5 | 5 | 5 | 5 | 5 | 5 | 3.66 | 4.71 | 5 | 4.57 |
| 8. I found this app very cumbersome/awkward to use. | 2 | 2 | 2 | 1 | 2 | 3 | 4 | 2 | 1 | 1 | 1 | 1 | 1 | 1 | 2 | 2 | 1 | 1.71 |
| 9. I felt very confident using this app. | 4 | 4 | 4 | 5 | 5 | 4 | 4 | 5 | 5 | 5 | 5 | 5 | 5 | 5 | 4 | 4.71 | 5 | 4.64 |
| 10. I needed to learn a lot of things before I could get going with this app. | 1 | 3 | 4 | 1 | 2 | 2 | 2 | 1 | 1 | 1 | 1 | 1 | 1 | 1 | 2.66 | 1.43 | 1 | 1.57 |

**Table 3. Total SUS scores and adjective ratings after first testing phase.**

| **Country** | **Total score** | **SUS score** | **Adjective rating** |
| --- | --- | --- | --- |
| **Estonia (n = 7)** | 34 | 85 | Excellent |
|  | 30 | 75 | Good |
|  | 29 | 72,5 | Good |
|  | 25 | 62,5 | Ok |
|  | 35 | 87,5 | Excellent |
|  | 39 | 97,5 | Excellent |
|  | 40 | 100 | Best imaginable |
| **Finland (n = 3)** | 26 | 65 | Ok |
|  | 27 | 67,5 | Ok |
|  | 24 | 60 | Ok |
| **Latvia (n = 4)** | 38 | 95 | Excellent |
|  | 38 | 95 | Excellent |
|  | 38 | 95 | Excellent |
|  | 38 | 95 | Excellent |

### Second phase of testing (version 2)

The overall average usability of the EmpowerKids tool in all countries was good, with an average SUS score of 84 across all countries (Tables 4 and 5). The professionals’ average system usability ratings were higher in Finland (95) and Latvia (92.5) than in Estonia (74). The professional in Finland (n = 1) rated the usability of the tool at 95 (excellent). For the professionals in Latvia (n = 4), the scores ranged from 85 (excellent) to 100 (best imaginable). For the professionals in Estonia (n = 5), the scores ranged from

62,5 (OK) to 80 (good).

**Table 4. SUS scores after second testing phase.**

| **Item** | FIN  (n =  1) |  | EE  (n = 5) | | |  |  | LV  (n = 4) | |  | Mean  (FIN) | Mean  (EE) | Mean  (LV) | Mean  (all) |
| --- | --- | --- | --- | --- | --- | --- | --- | --- | --- | --- | --- | --- | --- | --- |
| 1. I think that I would like to use this app frequently. | 3 | 4 | 3 | 4 | 3 | 4 | 4 | 5 | 5 | 5 | 3 | 3.6 | 4.75 | 4 |
| 2. I found this app unnecessarily complex. | 1 | 1 | 4 | 2 | 2 | 1 | 1 | 1 | 2 | 2 | 1 | 2 | 1.5 | 1.7 |
| 3. I thought this app was easy to use | 5 | 5 | 3 | 5 | 4 | 4 | 5 | 5 | 4 | 5 | 5 | 4.2 | 4.75 | 4.5 |
| 4. I think that I would need assistance to be | 1 | 1 | 1 | 1 | 2 | 1 | 1 | 1 | 1 | 2 | 1 | 1.2 | 1.25 | 1.2 |
| able to use this app. |  |  |  |  |  |  |  |  |  |  |  |  |  |  |
| 5. I found the various functions in this app were well integrated. | 5 | 4 | 5 | 4 | 3 | 3 | 5 | 5 | 4 | 4 | 5 | 3.8 | 4.5 | 4.2 |
| 6. I thought there was too much inconsistency in this app. | 1 | 3 | 4 | 2 | 3 | 3 | 2 | 1 | 1 | 1 | 1 | 3 | 1.25 | 2.1 |
| 7. I would imagine that most people would learn to use this app very quickly | 5 | 4 | 5 | 4 | 4 | 5 | 5 | 5 | 5 | 4 | 5 | 4.4 | 4.75 | 4.6 |
| 8. I found this app very cumbersome/awkward to use. | 1 | 4 | 4 | 2 | 3 | 2 | 1 | 1 | 1 | 1 | 1 | 3 | 1 | 2 |
| 9. I felt very confident using this app. | 5 | 5 | 5 | 5 | 3 | 4 | 5 | 5 | 4 | 4 | 5 | 4.4 | 4.5 | 4.5 |
| 10. I needed to learn a lot of things before I could get going with this app. | 1 | 1 | 1 | 3 | 2 | 1 | 1 | 1 | 1 | 2 | 1 | 1.6 | 1.25 | 1.4 |

**Table 5. Total SUS scores and adjective ratings after second testing phase.**

| **Country** | **Total score** | **SUS score** | **Adjective rating** |
| --- | --- | --- | --- |
| **Estonia (n=5)** | 32 | 80 | Good |
|  | 27 | 67,5 | Ok |
|  | 32 | 80 | Good |
|  | 25 | 62,5 | Ok |
|  | 32 | 80 | Good |
| **Finland (n=1)** | 38 | 95 | Excellent |
| **Latvia (n=4)** | 38 | 95 | Excellent |
|  | 40 | 100 | Best imaginable |
|  | 36 | 90 | Excellent |
|  | 34 | 85 | Excellent |

## Acceptability of the EmpowerKids tool

### First phase of testing

The overall acceptability of the tool was based on its suitability for the context

(children aged 6–12 years from low-income families, and professionals), satisfaction and quality, and attractiveness. The requirements for modification and other comments regarding the use of the tool were related to its overall coherence. The abbreviations (EE,

FI, LV) used in the following sections stand for Estonia (EE), Finland (FI) and Latvia (LV).

***Suitability for the context.*** The professionals considered that the tool was easy to use and simple to explain to children (EE, FI). The game design, graphics and music were also thought to be appropriate for children (EE, FI). In the professionals’ opinion, the child’s mood and desires (EE) and the current season (e.g., winter) might influence the child’s gameplay and response (EE, FI). Of the tool’s four games (physical activity, nutrition, daily rhythm and resources), the resources game was thought to be the easiest for a child to play independently. This was because children responded to the questions by choosing from four easy-to-understand “smiley faces.” The physical activity and nutrition games contained some graphs, which were thought to be irrelevant or confusing for players (EE, FI, LV). Furthermore, the professionals commented that the feedback after every section was too long and not tailored enough to a child’s individual needs (EE).

***Satisfaction and quality.*** The professionals in all three countries agreed that the tool was excellent for use in discussions with children. The concept of the tool was also appreciated, in that it offered professionals a new way to promote children’s health and wellbeing. The professionals valued its comprehensive overview of a child’s health and wellbeing was valued (EE, FI, LV). Technical difficulties, such as slowness and incorrect language options, were reported by professionals from one of the countries (EE). A few professionals also described the tool as time-consuming, due to all four sections having to be played at once (EE, FI).

***Attractiveness.*** The game graphics and music (birdsong) used in the tool was described as enjoyable and pleasant (EE, FI). The professionals expressed that children were eager to play the game and that they wanted to play it again later (EE). Some of the graphics were hard to understand (EE, FI); nevertheless, the professionals expressed that the tool was interesting for children to play and talk about afterwards (EE, LV).

***Modification needs.*** The professionals in all three countries were happy with the tool, but they made some suggestions for modification. They required more answer options in the daily rhythm game to cover choices such as the weekend and particular seasons (EE, FI, LV). Some of the professionals would have preferred simpler or fewer answer options in the physical activity and nutrition games (EE, FI). Professionals in every country expressed that the graphics should be clear enough for a child to understand (EE, FI, LV). In addition, they mentioned that capital letters would be a better option than lower-case letters for younger children (EE). The option to pause a single game and continue later would also have been appreciated (EE). Apart from these suggestions, one professional put forward the idea of including a “talking owl” to provide in-game help; this would enable the children to play some sections more independently, without needing constant assistance from an adult (FI).

### Second phase of testing

The EmpowerKids tool was modified in line with the relevant requirements that were observed in the first testing phase. The main modifications included adding more activity options for the daily rhythm game, adding the option to change the text to capital letters, and making it possible to play only one section at one time. In the second testing phase, the overall opinions regarding the acceptability of the tool were mostly concerned with the practicalities of using the tool in childcare settings.

***Suitability for the context***. All four sections of the EmpowerKids tool were found to be necessary for the professionals to be able to build an overall picture of a child’s health and wellbeing (EE, FI, LV). In addition, all the sections were important sources of information for detecting a child’s individual needs (EE, FI, LV). However, for the tool to be useful for a child, the child has to understand that they are meant to evaluate their current health status and health behavior. According to the professionals, this was a challenge for children of preschool age, some of whom answered according to what they wanted to be true or what they craved for. In addition, the large number of answer options to choose from in the tool as a whole, and the number of sports options in the physical activity section in particular, were thought to be exhausting for the children. This was because several alternatives had to be explained to children who had not experienced them or did not know what they were (EE, LV) or who found them confusing because of language barriers (FI). The professionals thought this was time-consuming (EE, LV). The professionals valued having discussions with a child during the gameplay, because this gave them an opportunity to talk with the child about health and wellbeing while they were playing and choosing alternatives EE). On the other hand, the professionals seemed to find discussion during the gameplay time-consuming and tiring for the younger children, whose attention sometimes wandered (EE, LV). The feature that made it possible to play the sections separately, and not necessarily in the same order, was an appreciated modification (LV).

***Satisfaction and quality***. All the professionals expressed that all four sections of the tool were needed (EE, FI, LV). The sections that received the highest satisfaction scores were daily rhythm (EE), nutrition (LV) and resources (EE. LV). The professionals appreciated the immediate feedback provided by the tool, which they could use in their discussions with a child about their health and wellbeing (EE). The tool was thought to be easy to use (FI) and a suitable method for use in individual conversations with children (EE). The tool was described as a useful method of working with children and as a resource that could tie in with various theme days (LV). There were some technical issues (such as slowness) in some parts of the tool (LV).

***Attractiveness***. The EmpowerKids tool was described as an interesting (LV), fun, enjoyable and cheerful (EE) application for children to use. The game design was described as easy and understandable, and the game graphics were described as attractive (FI). Using the app on a tablet was thought to be an easy way to start a discussion with the children in a different sort of way (LV).

***Modification needs***. The professionals suggested that the physical activity section could be modified by reducing or combining the sports options and by making this section shorter (EE, LV). They also suggested reducing the number of answer options in all sections, to improve understanding of the tool among children of preschool age (EE). Technical improvements regarding the speed and providing an alternative option for the appearance of the clock in the daily rhythm section were also mentioned (EE). In addition, it was suggested that the feedback section after every section could be more tailored to the individual child (FI).

# Discussion and conclusions

In this feasibility study, our aim was to explore the acceptability and usability of the EmpowerKids tool for professional users in various childcare and basic education settings. The findings of this study support the usability and acceptability of the tool in Estonia, Finland and Latvia. The results indicate that the usability of the tool is good and that the user experience is acceptable among professionals. The professionals perceived the tool as an excellent discussion resource to use with children. They valued the comprehensive overview provided of a child’s health and wellbeing for use in their discussions.

Children’s wellbeing encompasses quality of life in a broad sense [60]. It can be seen as a multi-dimensional construct that consists of mental, physical and social dimensions [15]. Approaching children’s wellbeing from a subjective perspective gives children an opportunity to express their own wellbeing and avoids making decisions in advance about which dimensions of life are important to children and the weights of the different dimensions [61]. The EmpowerKids tool aims to assess children’s health and wellbeing in different dimensions; when used as part of an intervention, it offers a feasible tool for professionals to use in order to have health discussions with children in a child-centered way. The tool not only focuses on the child’s challenges but also tries to identify the child’s strengths so professionals can draw on these to tackle the challenges detected as posing a risk to the child’s health and wellbeing [15, 62].

Games have been shown to have potential in health education for children, particularly in promoting physical activity and healthier dietary habits [33]; meanwhile, children perceive games as more useful and beneficial than other digital educational activities [63]. Schools offer one viable setting for health-promoting and game-based interventions. When implemented in a primary school context, a game-based intervention was shown to be effective in improving knowledge about mental health among 9- to 11-year-olds and encouraging them to put problems into perspective [64]. Another game-based intervention promoted positive attitudes and self-efficacy in relation to healthy eating among 10- to 11-year-olds [65]. However, these previous game-based interventions concentrated on a narrow dimension (mental vs physical) of a child’s wellbeing. Thus, the EmpowerKids tool is more multi-dimensional than previous game-based interventions, which calls for rigorous evaluation in school settings.

There is a need to monitor the wellbeing of children using systematic tools [62, 66]. The EmpowerKids tool offers one way of assessing the subjective wellbeing of children. Digital wellbeing assessment tools enable fluent and real-time data collection, alongside analyses and longitudinal monitoring of users’ health data; they also offer an easy access database for professionals, organizations and decision makers to use in order to guide the planning of targeted and needs-based interventions and services for children [67]. There are few existing validated web-based tools for assessing children’s wellbeing, but being more “structured query” type measures [62], they differ from the EmpowerKids tool, which offers a more subjective overview of a child’s wellbeing for use in health discussions. Thus, more research is needed before the data from the tool can be used as valid measurements and as a database that can inform, for example, decision makers.

The EmpowerKids tool is promising in terms of detecting a child’s individual health and wellbeing needs. It is a useful resource for professionals in discussions about children’s health behaviors, their overall wellbeing and their support needs. The tool is most suitable for children of school age; children of preschool age and children with cognitive impairments are likely to need more assistance to understand the concept and content of the tool. In addition, the low socioeconomic status and insufficient previous knowledge of the children who participated in this study might have influenced their understanding of some of the content, as indicated by the professionals. Thus, the tool should be modified further to fit better with every socioeconomic context and support the health literacy of children and families from lower socioeconomic groups.

One of the strengths of this feasibility study was the data collection methods, which included quantitative and qualitative assessments of the feasibility of the EmpowerKids tool. The usability and acceptability were measured twice in diverse contexts in three different countries. Furthermore, the selected measure for assessing usability has been widely used and is considered valid [54, 68]. This study focused on the perceptions of the professionals. Even though the professionals also reflected on the perceptions of children in their answers, it would be useful to evaluate the feasibility of the tool from the perspective of children. It would also be meaningful to evaluate the effectiveness of the intervention from the perspective of child outcomes, especially before planning its implementation [46].

There were also some limitations to this study. The sample size and sampling method were restricted to professionals who were working on the EmpowerKids project. However, in comparison with colleagues outside the project, the chosen study participants might have had a stronger interest in developing methods for professionals working on health and wellbeing with children from low-income families. Nevertheless, the

generalizability of the results is limited.

The results of this feasibility study suggest that the EmpowerKids tool is feasible in several childcare and education settings in Estonia, Finland and Latvia. Children’s age and level of cognitive understanding, professional users’ previous knowledge of each child, and suitable education and training in using the tool are important factors to consider when using this method with children. When used as part of an intervention, the tool offers a promising method of identifying a child’s individual circumstances and needs in order to promote their health and wellbeing.

# Acknowledgments

We would like to thank our colleagues at the EmpowerKids project for their support in planning, organizing and implementing the study in the three countries. We also express our profound gratitude to the children, and to the study participants, for their time and generous feedback.

# References

1. Pavalko E, Caputo J. Social Inequality and Health Across the Life Course. American

Behavioral Scientist. 2013;57(8):1040-1056.

2. Allin S, Stabile M. Socioeconomic Status and Child Health: What is the Role of Health Care, Health Conditions, Injuries and Maternal Health? Health Econ Policy Law. 2012;7 (2): 227-242.
3. Kruk K. Parental income and the dynamics of health inequality in early childhood evidence from the UK. Health Economics. 2012;22(10):1199-1214.
4. Kutner M. The health literacy of America's adults. Washington, D.C.: U.S. Dept. of Education, National Center for Education Statistics; 2006.
5. Rikard RV, Thompson MS, McKinney J, Beauchamp A. Examining Health Literacy

Disparities in the United States: A Third Look at the National Assessment of Adult

Literacy (NAAL). BMC Public Health. 2016;16: 975.

6. Bröder J, Okan O, Bauer U, Bruland D, Schlupp S, Bollweg T et al. Health literacy in childhood and youth: a systematic review of definitions and models. BMC Public

Health. 2017;17(1).

7. Nielsen-Bohlman L. Health literacy. Washington, D.C.: National Academies Press;

2004.

8. Sørensen K, Van den Broucke S, Fullam J, Doyle G, Pelikan J, Slonska Z et al. Health literacy and public health: A systematic review and integration of definitions and models. BMC Public Health. 2012;12(1).
9. Taggart J, Williams A, Dennis S, Newall A, Shortus T, Zwar N et al. A systematic review of interventions in primary care to improve health literacy for chronic disease behavioral risk factors. BMC Family Practice. 2012;13(1).
10. Jorm AF, Korten AE, Rodgers B, Pollitt P, Jacomb PA, Christensen H. Belief systems of the general public concerning the appropriate treatments for mental disorders. Soc Psychiatry Psychiatr Epidemiol. 1997;32: 468-73.
11. Tully L, Hawes D, Doyle F, Sawyer M, Dadds M. A national child mental health literacy initiative is needed to reduce childhood mental health disorders. Australian & New Zealand Journal of Psychiatry. 2019;53(4):286-290.
12. Riebschleger J, Costello S, Cavanaugh DL, Grové C. Mental health literacy of youth that have a family member with a mental illness: Outcomes from a new program and scale. Frontiers in psychiatry. 2019;10(2).
13. Berkman ND, Sheridan SL, Donahue KE, Halpern DJ, Crotty K. Low Health Literacy and Health Outcomes: An Updated Systematic Review. Ann Intern Med.

2011;155: 97-107.

14. United Nations. Convention on the Rights of the Child. General Assembly

Resolution 44/25; 1989.

15. Pollard E, Lee P. Child Well-Being: A Systematic Review of the Literature. Social

Indicators Research. 2003;61: 59–78.

16. Landry B, Driscoll S. Physical Activity in Children and Adolescents. PM&R.

2012;4(11):826-832.

17. Engle P, Huffman S. Growing Children's Bodies and Minds: Maximizing Child

Nutrition and Development. Food and Nutrition Bulletin. 2010;31(2_suppl2):S186S197.

18. Chien N, Mistry R. Geographic Variations in Cost of Living: Associations With Family and Child Well-Being. Child Development. 2012;84(1):209-225.
19. Conger R, Conger K, Martin M. Socioeconomic Status, Family Processes,and

Individual Development. Journal of Marriage and Family. 2010;72(3):685-704.

20. Bray M, Young M. Circadian rhythms in the development of obesity: potential role for the circadian clock within the adipocyte. Obesity Reviews. 2007;8(2):169-181.
21. Sevón E, Rönkä A, Räikkönen E, Laitinen N. Daily rhythms of young children in the 24/7 economy: A comparison of children in day care and day and night care. Childhood. 2017;24(4):453-469.
22. Hertzman C, Williams R. Making early childhood count. Canadian Medical
23. Bleich SN, Vercammen KA, Zatz LY, Frelier JM, Ebbeling CB, Peeters A.

Interventions to prevent global childhood overweight and obesity: a systematic review.

Lancet Diabetes Endocrinol. 2018;6 (4): 332-346.

24. Nagata J. New Findings From the Health Behaviour in School-aged Children (HBSC) Survey: Social Media, Social Determinants, and Mental Health. Journal of Adolescent Health. 2020;66(6):S1-S2.
25. Mediterranean W. Health education: theoretical concepts, effective strategies and core competencies: a foundation document to guide capacity development of health educators [Internet]. Apps.who.int. 2021 [cited 23 September 2021]. Available from: <https://apps.who.int/iris/handle/10665/119953?show=full>
26. WHO. Health Education: Theoretical Concepts, Effective Strategies and Core

Competencies: A Foundation Document to Guide Capacity Development of Health Educators 2012. [cited 23 September 2021]. Available from:

[http://applications.emro.who.int/dsaf/EMRPUB_2012_EN_1362.pdf.](http://applications.emro.who.int/dsaf/EMRPUB_2012_EN_1362.pdf)

27. WHO. The Ottawa Charter for Health Promotion. Milestones in Health Promotion:

Statements from Global Conferences. Geneva: World Health Organization 2009. [cited 23 September 2021]. Available from: [https://www.who.int/healthpromotion/Milestones_Health_Promotion_05022010.pdf.](https://www.who.int/healthpromotion/Milestones_Health_Promotion_05022010.pdf)

28. Darling-Hammond L, Flook L, Cook-Harvey C, Barron B, Osher D. Implications for educational practice of the science of learning and development. Applied

Developmental Science. 2019;24(2):97-140.

Association Journal. 2009;180(1):68-71.

29. Sanders L, Shaw J, Guez G, Baur C, Rudd R. Health Literacy and Child Health

Promotion: Implications for Research, Clinical Care, and Public Policy. Pediatrics.

2009;124(Supplement 3):S306-S314.

30. O’Reilly M, Svirydzenka N, Adams S, Dogra N. Review of mental health promotion interventions in schools. Social Psychiatry and Psychiatric Epidemiology.

2018;53(7):647-662.

31. Jacob C, Hardy-Johnson P, Inskip H, Morris T, Parsons C, Barrett M et al. A systematic review and meta-analysis of school-based interventions with health education to reduce body mass index in adolescents aged 10 to 19 years. International Journal of Behavioral Nutrition and Physical Activity. 2021;18(1).
32. Ledoux T, Griffith M, Thompson D, Nguyen N, Watson K, Baranowski J et al. An

Educational Video Game for Nutrition of Young People. Simulation & Gaming.

2016;47(4):490-516.

33. Parisod H, Pakarinen A, Kauhanen L, Aromaa M, Leppänen V, Liukkonen T et al.

Promoting Children's Health with Digital Games: A Review of Reviews. Games for Health Journal. 2014;3(3):145-156.

34. Adams SA. Use of “Serious Health Games” in Health Care: A Review. Stud Health Technol Inform. 2010;157: 160–166.
35. Lustria M, Cortese J, Noar S, Glueckauf R. Computer-tailored health interventions delivered over the web: Review and analysis of key components. Patient Education and Counseling. 2009;74(2):156-173.
36. Johnson D, Deterding S, Kuhn K, Staneva A, Stoyanov S, Hides L. Gamification for health and wellbeing: A systematic review of the literature. Internet Interventions.

2016;6:89-106.

37. Charlier N, Zupancic N, Fieuws S, Denhaerynck K, Zaman B, Moons P. Serious games for improving knowledge and self-management in young people with chronic conditions: a systematic review and meta-analysis. Journal of the American Medical Informatics Association. 2015;23(1):230-239.
38. Lau H, Smit J, Fleming T, Riper H. Serious Games for Mental Health: Are They Accessible, Feasible, and Effective? A Systematic Review and Meta-analysis. Frontiers in Psychiatry. 2017;7.
39. DeSmet A, Van Ryckeghem D, Compernolle S, Baranowski T, Thompson D, Crombez G et al. A meta-analysis of serious digital games for healthy lifestyle promotion. Preventive Medicine. 2014;69:95-107.
40. Fernandez-Luque L, Tøllefsen T, Brox E. Healthy Gaming – Video Game Design to promote Health. Applied Clinical Informatics. 2011;02(02):128-142.
41. Parisod H, Pakarinen A, Kauhanen L, Aromaa M, Leppänen V, Liukkonen TN et al.

The advantages and limitations of digital games in children’s health promotion. Finnish Journal of eHealth and eWelfare. 2014;6(4): 164–173.

42. EmpowerKids. About EmpowerKids Project. Empowerkids. [cited 20 July 2020]. EmpowerKids 2017. Available from: [http://www.empowerkids.eu/content/about-](http://www.empowerkids.eu/content/about-project)

[project.](http://www.empowerkids.eu/content/about-project)

43. Pakarinen A, Mackiewicz K. Games can help empower children from low-income families to take healthy choices in daily life. European Journal of Public Health.

2017;27(suppl_3).

44. Barnum CM. Usability Testing Essentials: Ready, Set…Test! Amsterdam: Elsevier; 2010.
45. Bowen D, Kreuter M, Spring B, Cofta-Woerpel L, Linnan L, Weiner D et al. How We Design Feasibility Studies. Am J Prev Med 2009;36 (5): 452-457.
46. Craig P, Dieppe P, Macintyre S, Michie S, Nazareth I, Petticrew M. Developing and evaluating complex interventions: the new Medical Research Council guidance. BMJ.

2008;:a1655.

47. Grove S, Burns N, Gray J. The practice of nursing research. The practice of nursing research: Appraisal, synthesis, and generation of evidence. Elsevier Health Sciences; 2013.
48. Lou C, Anthony E, Stone S, Vu C, Austin M. Assessing Child and Youth WeilBeing. Journal of Evidence-Based Social Work. 2008;5(1-2):91-133.
49. Statham J, Chase E. Childhood Wellbeing: A Brief Overview. Loughborough:

Childhood Wellbeing Research Centre; 2010.

50. Dijkstra T. Child Well-Being in Rich Countries: UNICEF’s Ranking Revisited, and

New Symmetric Aggregating Operators Exemplified. Child Indicators Research.

2009;2(3):303-317.

51. WHO. Global recommendations on physical activity for health. .. Geneva: World Health Organization; 2010.
52. Arain M, Campbell MJ, Cooper CL, Lancaster GA. What is a pilot or feasibility study? A review of current practice and editorial policy. BMC Med. Res. Methodol.

2010;10 (67): 1-7.

53. Shanyinde M, Pickering R, Weatherall M. Questions asked and answered in pilot and feasibility randomized controlled trials. BMC Medical Research Methodology. 2011;11(1).
54. Brooke J. System Usability Scale (SUS): A Quick-and-Dirty Method of System

Evaluation User Information. UK: Digital Equipment Co Ltd; 1996.

55. Brooke J. SUS: A Retrospective. J Usability stud. 2013;8 (2): 29-40.
56. Bangor A, Kortum P, Miller J. Determining what individual SUS scores mean: Adding an adjective rating scale. J Usability stud. 2009;4 (3): 114-123.
57. Elo S, Kääriäinen M, Kanste O, Pölkki T, Utriainen K, Kyngäs H. Qualitative content analysis: A focus on trustworthiness. SAGE open. 2014:4(1).
58. World Medical Association Declaration of Helsinki. JAMA. 2013;310(20):2191.
59. Shamoo A, Resnik D. Responsible conduct of research. Oxford University Press;

2009.

60. Ben-Arieh A, Frønes I. Indicators of Children’s Well-being: What should be Measured and Why? Soc Indic Res. 2007;84: 249-250.
61. Bradshaw J. Child well-being in comparative perspective. Children Australia.

2009;34(1):5-13.

62. Rosanbalm K, Snyder E, Lawrence C, Coleman K, Frey J, van den Ende J et al. Child wellbeing assessment in child welfare: A review of four measures. Children and

Youth Services Review. 2016;68:1-16.

63. Gigantesco A, Palumbo G, Zadworna-Cieślak M, Cascavilla I, Del Re D,

Kossakowska K. An international study of middle school students’ preferences about digital interactive education activities for promoting psychological well-being and mental health. Annali dell'Istituto superiore di sanita. 2019;55(2): 108-117.

64. Shum AK, Lai ES, Leung WG, Cheng MN, Wong HK, So SW et al. A digital game and school-based intervention for students in Hong Kong: quasi-experimental design.

Journal of medical Internet research. 2019;21(4): e12003.

65. Schneider KL, Ferrara J, Lance B, Karetas A, Druker S, Panza E et al. Acceptability of an online health videogame to improve diet and physical activity in elementary school students: “Fitter Critters”. Games for Health: Research, Development, and

Clinical Applications. 2012;1(4): 262-268.

66. Cho EYN, Yu FYA review of measurement tools for child wellbeing. Children and Youth Services Review. 2021;105576.
67. Gay V, Leijdekkers P. Bringing Health and Fitness Data Together for Connected Health Care: Mobile Apps as Enablers of Interoperability. Journal of Medical Internet

Research. 2015;17(11):e260.

68. Burke PF. Seeking simplicity in complexity: The relative value of ease of use

(EOU)‐based product differentiation. Journal of Product Innovation Management, 2013;30(6): 1227-1241.
